# Advancing Periodontal Care: Development of a Novel Collagen-Chitosan-Bioglass Scaffold as a Substitute for Autologous Soft Tissue Grafts

**DOI:** 10.7759/cureus.68644

**Published:** 2024-09-04

**Authors:** Hemaanhini Tamilmani, B Kiran Srinivas, Nidhita Suresh, K Saranya

**Affiliations:** 1 Periodontology, Saveetha Dental College and Hospitals, Saveetha Institute of Medical and Technical Sciences, Saveetha University, Chennai, IND

**Keywords:** aesthetics, chitosan, collagen, bioglass, gingival recession

## Abstract

Introduction

Modern dentistry prioritizes aesthetic outcomes, making root coverage for gingival recession a key focus. Various approaches, including autologous grafts, address this issue, yet no substitute matches the properties of autogenous connective tissue grafts. The innovative collagen-chitosan-bioglass scaffold presents a promising solution, surpassing the limitations of the traditional methods. This scaffold blends the advantages of collagen with chitosan's antibacterial and regenerative properties, enhanced by bioglass, which promotes tissue healing through angiogenesis. It was evaluated for its physicochemical characteristics, as well as antioxidative and anti-inflammatory properties, making it a promising solution for soft tissue management in dentistry.

Materials and methods

Chitosan, collagen, and bioglass were combined into a scaffold through the lyophilization process (freeze-drying). Chitosan was sourced from shrimp, collagen from bovine, and the bioglass 1% comprised 58% tetra-ethyl ortho silicate, 33% calcium silicate, and phosphorous pentoxide. After the scaffold was created, it was subjected to physicochemical characterization via scanning electron microscopic and infrared spectroscopic analysis. Its anti-inflammatory and antioxidant properties were evaluated using DPPH (2,2-diphenyl -1-picrylhydrazyl) assay and by measuring the scaffold's radical scavenging activity.

Results

This study employed infrared spectroscopy and scanning electron microscopy techniques to analyze the sample components and their morphology. The infrared (attenuated total reflection) analysis revealed various elements confirming the presence of all the biomaterials required to fabricate the scaffold. Scanning electron microscope imaging displayed a folded-like morphology with a porous structure. The protein denaturation inhibition increased from 25% at 50 μg of scaffold weight to 45% at 200 μg of scaffold weight. Similarly, the antioxidant activity increased, with values rising from 23% at 50μg to 35% at 200μg of scaffold weight.

Conclusion

The fabricated collagen-chitosan-bioglass scaffold demonstrates promising antioxidant and anti-inflammatory properties. These findings suggest that this scaffold holds significant potential as a viable substitute for soft tissue augmentation.

## Introduction

Modern dentistry increasingly prioritizes the patient's aesthetic expectations, with root coverage for gingival recession becoming a fundamental aspect of aesthetics and periodontics. Gingival recession is defined by the apical migration of gingival tissues away from the cementoenamel junction, leading to the exposure of root surfaces [1]. Multiple approaches have been employed to manage gingival recession, such as pedicle flaps, tunneling, and combination procedures along with the placement of autologous grafts and soft tissue substitutes. Until now, there is no comparable substitute for autogenous connective tissue graft in terms of its properties, predictability, and long-term outcomes. While connective tissue grafts remain the gold standard, utilizing alternative grafting materials offers distinct advantages, including diminished morbidity, shortened surgical duration, and unlimited supply.

Engineered to surpass the limitations of these autologous soft tissue grafts, the innovative collagen-chitosan-bioglass scaffold holds immense promise for effectively managing gingival recession. This revolutionary approach aims to address the challenges associated with traditional methods, providing enhanced effectiveness and patient satisfaction. The soft tissue substitute comprises a collagen-chitosan-bioglass network, combining the benefits of collagen's lower immunogenicity, non-toxicity, biodegradability, and biocompatibility compared to other polymers [2]. The scaffold can be blended with different materials to form a range of practical scaffolds [3]. Chitosan, a natural polysaccharide biopolymer derived from chitin, the primary structural component of crustacean exoskeletons, undergoes chemical or enzymatic deacetylation to form chitosan [4,5]. In the physiological environment, the unique properties of chitosan arise from the polycationic nature of its protonated amino groups. Due to electrostatic forces, this polycationic nature enables chitosan molecules to disrupt the negatively charged cell walls of bacteria [6].

Due to its demonstrated mucoadhesive and hemostatic properties, incorporating chitosan into a porous collagen matrix generally enhances the material's overall healing and antibacterial characteristics [7]. The antibacterial efficacy of chitosan, attributed to its polycationic nature that interacts preferentially with negatively charged microbial cell walls and cytoplasmic membranes, leads to reduced osmotic stability, membrane rupture, and eventual leakage of intracellular components [8]. As a biocompatible and biodegradable polysaccharide derived from chitin in crustacean exoskeletons, such as shrimp and crabs, chitosan has garnered attention in regenerative medicine. Its unique attributes, including the formation of scaffolds supporting cell growth and tissue regeneration, contribute to its significant role in this field [9]. Moreover, researchers have explored chitosan's antibacterial potential, positioning it as a promising substance for regenerative medicine applications, particularly in scenarios demanding stringent infection control. Chitosan can penetrate bacterial and fungal nuclei, binding to microbial deoxyribonucleic acid to impede messenger ribonucleic acid and protein synthesis. Furthermore, chitosan has effectively inhibited bacterial biofilm formation-resilient communities encased in a protective extracellular matrix, rendering them more resistant to antibiotics [10].

Bioglass, a bioactive compound and a silica-based regenerative material, is known for its biocompatibility, bioactivity, and osteoconductive properties, which can act as an excellent regenerative material [11]. It stands out among inorganic materials for its extensive exploration of soft tissue regeneration. This material has found applications in cardiac, lung, nervous, and epithelial tissue studies and has proven to be a promising candidate for treating soft tissue damage [12]. Bioglass contributes to all four stages of wound healing, fostering the acceleration of skin repair and regeneration. This involves heightened cell proliferation, diminished inflammation, enhanced angiogenesis, and antibacterial effects attributed to bioglass particles at the injury site [13]. Hence, in this present study, we have incorporated bioglass into the collagen-chitosan scaffold and evaluated for the physicochemical characterization, anti-inflammatory, and antioxidative efficacy of collagen-chitosan-bioglass scaffold.

## Materials and methods

The collagen-chitosan-bioglass scaffold was fabricated by utilizing chitosan (Himedia, Thane, India) derived from shrimp, while collagen (Himedia) was sourced from bovine. Bioglass 1% [14], consisting of 58% tetra-ethyl ortho silicate, 33% calcium silicate, and phosphorous pentoxide, was combined with 3g of chitosan and 3g of collagen through lyophilization (freeze-drying) to form a matrix. This process involves removing water molecules by freeze-drying to prevent coagulation and loss of properties in proteins, which cannot be heat-dried. The matrix, formed by lyophilizing a combination of chitosan, collagen, and bioglass, was evaluated for its physical properties through scanning electron microscopy.

Surface morphology and topography were evaluated through scanning electron microscopy (field emission scanning electron microscopy, JEOL JSM IT800, JEOL Ltd., Tokyo, Japan), utilizing a high-energy beam for backscattering electrons, with the characteristics of X-rays recorded and converted into images by electron detectors. To analyze the chemical composition of the collagen-chitosan bioglass scaffold, Fourier transform infrared (FTIR) spectra were obtained using a Bruker Alpha II instrument equipped with attenuated total reflectance (ATR) sampling (Bruker, Billerica, MA, USA).

The antioxidant activity was assessed by scavenging free radicals, which can bind to the charged molecules of bacteria and eliminate them [15]. The radical scavenging activity was determined using the 2,2-diphenyl-1-picrylhydrazyl assay, where a 0.1 mM 2,2-diphenyl-1-picrylhydrazyl solution was prepared by dissolving 4 mg of 2,2-diphenyl-1-picrylhydrazyl in 100 ml of ethanol. The absorption decrease of the 2,2-diphenyl-1-picrylhydrazyl solution after adding the sample (50 μg and 200 μg) was measured at 517 nm. Ascorbic acid (10mg/ml dimethyl sulfoxide) served as a reference. The percentage of radical scavenging activity was calculated using the following formula:

$\%RSA = \left( \frac{{\text{Abs}_{\text{control}} - \text{Abs}_{\text{sample}}}}{{\text{Abs}_{\text{control}}}} \right) \times 100$

where RSA represents the radical scavenging activity, Abs_control_ represents the absorbance value of the control, and Abs_sample_ the absorbance value of the sample.

A 2% solution prepared with 0.05 M Tris HCI containing 1.5 ml of bovine serum albumin was mixed with different extract concentrations (50 and 200 mg/mL). To change the pH of the finished solution, Tris HCl was utilized. After 30 minutes of incubation, the samples were prepared and immersed in water, which was heated to 75°C for 10 minutes. Afterward, the samples were allowed to cool down to room temperature. Using an ultraviolet spectrophotometer set at 660 nm, the turbidity of the samples was measured, and the percentage inhibition of albumin denaturation was calculated using the following equation:

$\text{Anti-inflammatory (\%)} = \frac{\text{Optical density}_{\text{control}} - \text{Optical density}_{\text{sample}}}{\text{Optical density}_{\text{control}}} \times 100$

where Optical density_control_ and Optical density_sample_ represent the optical density values of the control and the sample, respectively.

## Results

**Figure 1:**
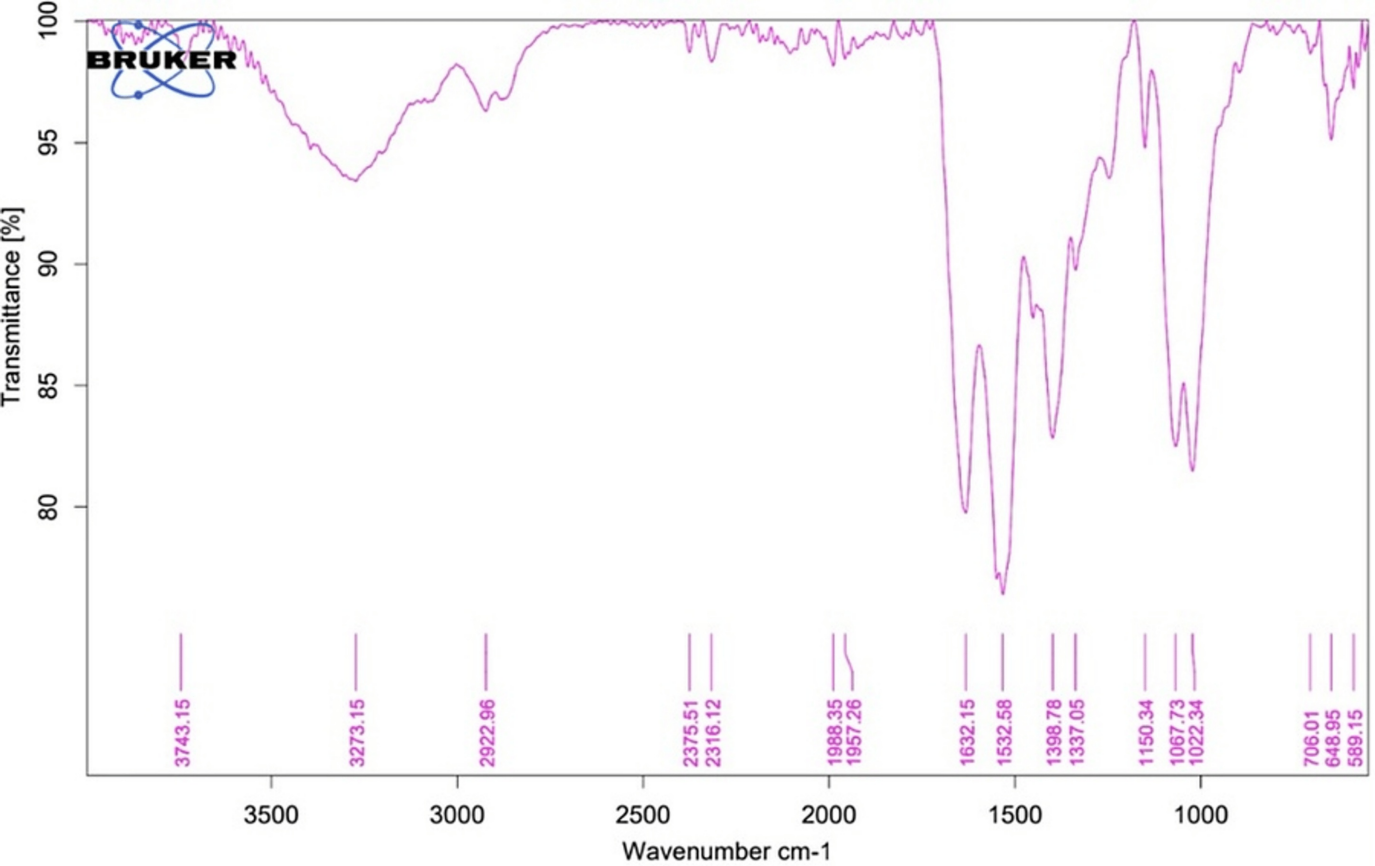
Infrared spectrum (attenuated total reflection) of the collagen-chitosan-bioglass scaffold

**Figure 2:**
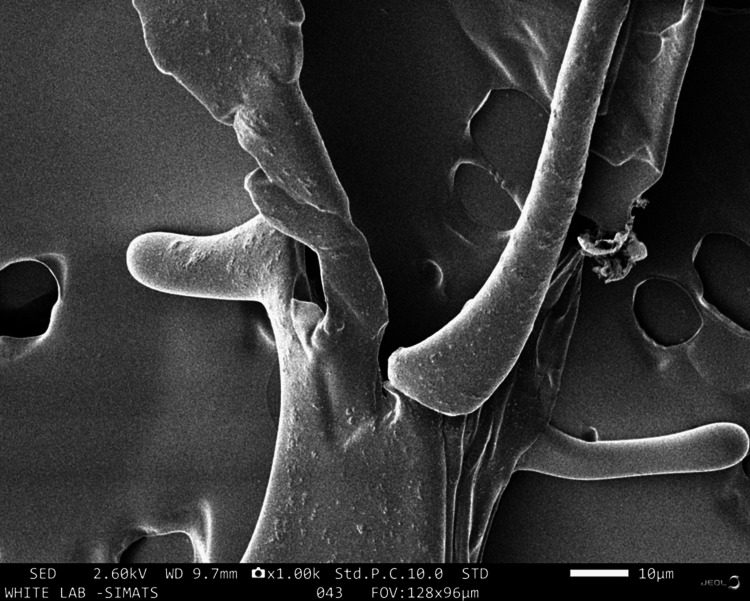
Scanning electron microscopic image of the collagen-chitosan-bioglass scaffold

**Figure 3:**
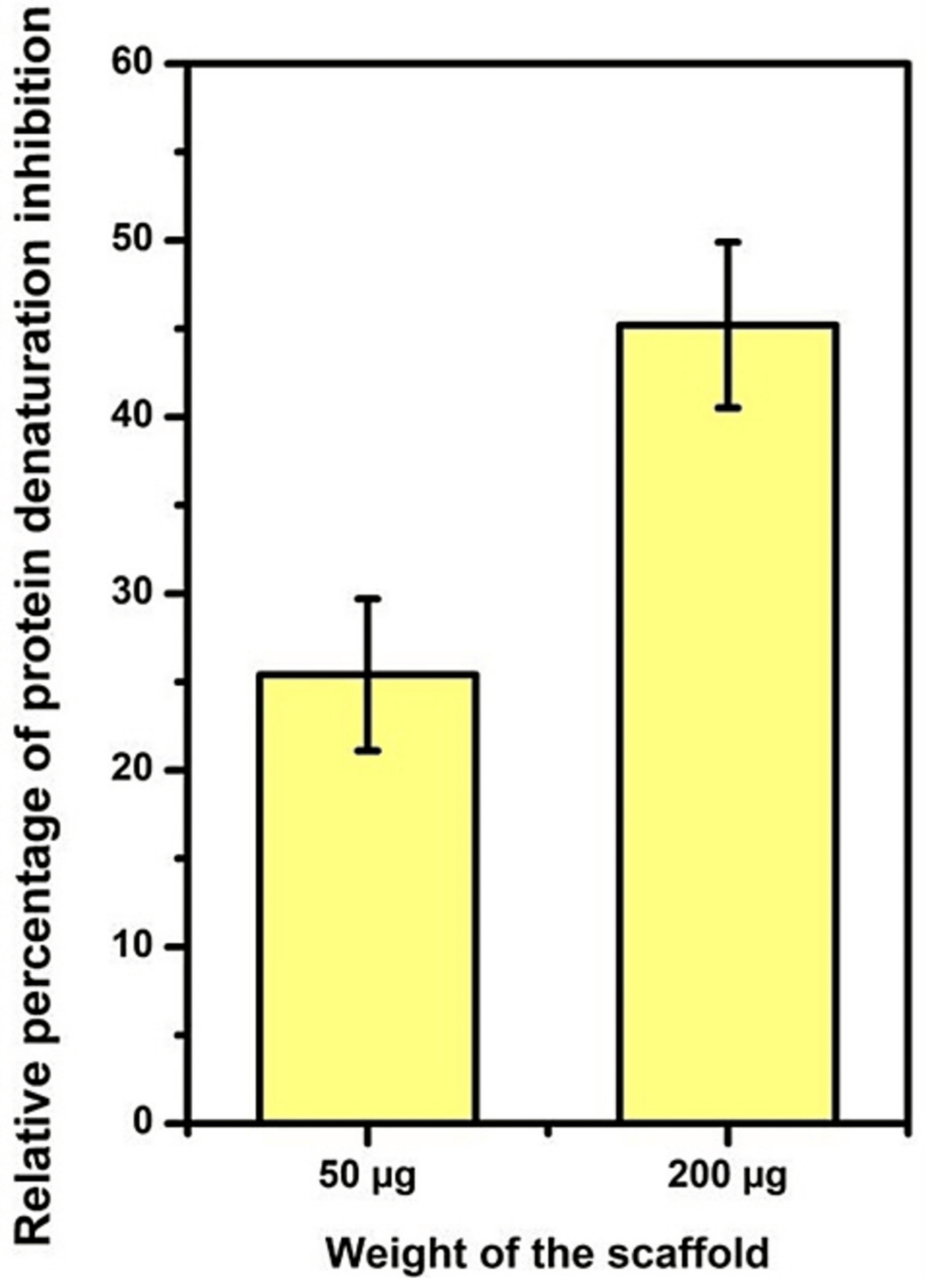
Anti-inflammatory activity of the collagen-chitosan-bioglass scaffold

This study utilized infrared (IR) spectroscopy (attenuated total reflection) and scanning electron microscopy (SEM) techniques to analyze the components and morphology of the sample. The IR analysis revealed various elements within the matrix at different wavelengths: OH group at 3273 nm, CH_3_ at 2922 nm, and N-H group at 1632 nm, indicating the presence of chitosan. Additionally, the presence of amide I was confirmed at 1632 nm. Other constituents detected include amide II at 1532 nm, pyrrolidine rings at 1398 nm, C-O-N at 1337 nm, glycosidic bonding at 1150 nm, and C-O group at 1067 nm, indicating the presence of collagen as shown in Figure 1 and Table 1. The SEM image in Figure 2 reveals a folded-like morphology with a porous structure. Figure 3 depicts the relationship between the weight of the collagen-chitosan-bioglass framework and its anti-inflammatory activity, showing that as the weight increases, there is a proportional rise in the percentage of inhibition of protein denaturation. The inhibition of protein denaturation for the fabricated scaffold at 50μg and 200μg was measured to be 25% and 45%, respectively.

**Table 1:** Peaks observed (reported as wavenumbers) in the infrared (attenuated total reflection) spectrum of the collagen-chitosan-bioglass scaffold

Peak at wavelength	Element	Component
3273	OH group	Chitosan
2922	CH3	Chitosan
1632	N-H Group	Chitosan
-	N-H Group	Amide I
1532	Amide II	Collagen
1398	Pyrrolidine rings	Collagen
1337	C-O-N	Chitosan
1150	Glycosidic bonding	Chitosan
1067	C-O group	Collagen

**Figure 4:**
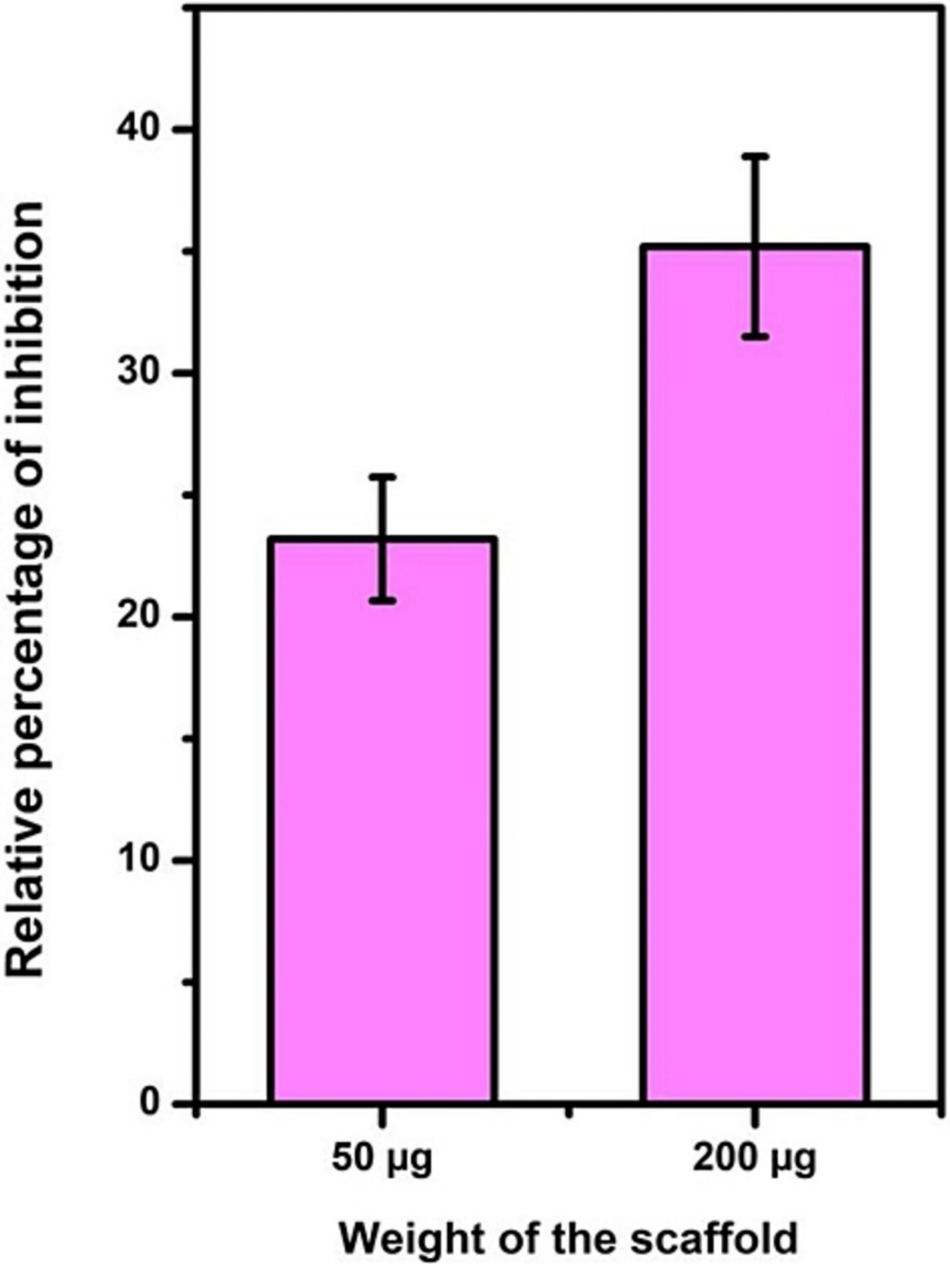
Antioxidant activity of the collagen-chitosan-bioglass scaffold

Figure 4 illustrates the antioxidant activity, demonstrating that as the scaffold weight increased from 50μg to 200μg, the percentage of inhibition rose from 23% to 35%. In comparison, the control exhibited 100% inhibition.

## Discussion

Collagen's exceptional properties, including its mechanical strength, hemostatic capability, low antigenicity, non-cytotoxicity, biocompatibility, biodegradability, hydrophilicity, and strong cell attachment affinity, make it a preferred choice for scaffold fabrication [16]. Chitosan cryogels facilitate macrophage proliferation and self-adhesion, rendering them versatile for various applications. However, pure dried chitosan scaffolds lack sufficient mechanical stability and bioactivity, prompting their combination with other polymers to augment their characteristics [17]. Different fabrication methods, such as salt leaching, electrospinning, freezing, and lyophilization, are utilized to create dried scaffolds [18]. In our research, we have developed a collagen-chitosan-bioglass matrix for soft tissue regeneration using lyophilization.

The survival rates of teeth and dental implants have increased using soft tissue augmentation techniques to correct mucogingival abnormalities and provide functional and aesthetic results. Gingival tissue dimension has been increased by using numerous surgical procedures [19]. These procedures can be divided into two primary groups: those that create and broaden keratinized mucosa and those that increase the volume/thickness of soft tissue. Autogenous connective tissue grafts are currently the gold standard, regardless of the technique employed. Because a donor location is needed for this autologous transplant, there will be more post-operative pain and procedure time morbidity [20]. Using an autograft taken from the palate suggests potential side effects, including bleeding from injury to the palatine artery's branches, necrosis of the mucosa, and anesthesia. Biomaterials are being used as substitutes to prevent these issues associated with the second surgical site. Based on where they come from, these substitute products can be roughly categorized into three groups: synthetic (alloplastic), xenogeneic, and allogeneic materials [20,21]. There may be moral dilemmas and a chance of disease transmission when using an acellular dermal matrix allograft made from human cadaveric skin. After being treated to eliminate antigenic cellular components, xenogeneic pig collagen matrices have been touted as an infinite substitute for connective tissue grafts and acellular dermal matrix, yielding outcomes similar to autogenous soft tissue constructs. These biomaterial substitutes function as a three-dimensional scaffold, enabling the constant synthesis of new connective tissue and the breakdown of the original matrix.

Many soft tissue substitutes are routinely used, such as Fibro-Gide (Geistlich Pharma AG, Wolhusen, Switzerland), which helps ensure superior tissue volume stability as its primary benefit. This collagen matrix goes through cross-linking, which gives it some elasticity [22]. Thus, several physical, biological, and chemical cross-linking techniques have been used to reinforce the mechanical and biodegradable stabilities, producing cross-linked collagen in general. This increased the tensile strength and a more extended period before collagen broke down [23]. 

Other substitutes, such as Alloderm, have recently gained attention in repairing and regenerating periodontal and gingival tissues. The palatal donor sites have recently been replaced with an acellular dermal matrix graft to widen the keratinized tissue around implants and teeth, repair abnormalities of the alveolar ridge, and perform root-covering treatments [24]. After processing the dermis from a human donor, all cells are removed, leaving a type-I collagen-based connective tissue matrix that is structurally intact. Harris [24] reported that an acellular dermal matrix graft with a flap was used coronally to treat gingival recession. Consistently integrating into the host tissue, the acellular dermal matrix preserved the tissue's structural integrity and allowed for revascularization through the preservation of vascular channels. It was also noted that the achieved color match was similar to the connective tissue graft [25].

Incorporating bioactive glass can degrade at a controllable rate and convert to a hydroxyapatite (HA)-like material. This material bonds firmly to hard and soft tissues and releases ions during the degradation process. These ions are known to have a beneficial effect on osteogenesis and angiogenesis [26]. They can also increase the fabricated matrix's physicochemical properties. This fabricated collagen-chitosan-bioglass matrix also possesses a unique antimicrobial property against gram-positive and gram-negative microorganisms [27]. The limitations of the study are not analyzing the degradation and antibacterial efficacy of the collagen-chitosan-bioglass scaffold. Furthermore, its commercial use has to be explored by conducting in vivo and clinical trials.

## Conclusions

The development of the innovative collagen-chitosan-bioglass scaffold stands out as an optimal choice for tissue regeneration due to its inherent anti-inflammatory and antioxidant properties, as well as its composition of biomaterials that are well-established in promoting tissue healing and regeneration. It holds potential as a viable alternative to autologous grafts for soft tissue regeneration in the future.
